# How does workplace ostracism affect employee innovation behavior: An analysis of chain mediating effect

**DOI:** 10.3389/fpsyg.2022.920914

**Published:** 2022-08-17

**Authors:** Yimeng Xing, Yongzhou Li

**Affiliations:** Center For Industrial Policy and Management Research, Evergrande School of Management, Wuhan University of Science and Technology, Wuhan, China

**Keywords:** workplace ostracism, knowledge hiding, organizational identification, innovation behavior, task interdependence

## Abstract

This study seeks to examine the relationship between workplace ostracism and innovation behavior while considering the mediating role of knowledge hiding and organizational identification. The study also tests the moderating role of task interdependence in these relationships. The study collected data through structured questionnaires from 409 participants (i.e., employees) working in the small to medium-sized enterprise of big cities of China. The study adopted a structured equation modeling technique for data analysis. Significantly, the study results suggest that workplace ostracism is negatively associated with innovation behavior, both directly and indirectly *via* knowledge hiding and organizational identification. We also find that task interdependence weakens the positive relationship between workplace ostracism and knowledge hiding. Current study has tested the negative relationship between workplace ostracism and innovation behavior unlike most of the previous investigations that have focused on positive factors. Our study from a rational perspective to explore the influence mechanism between workplace ostracism and innovation behavior is addition to the previous research and the rich, in revelation managers motivate employees to implement knowledge sharing activities at the same time, pay attention to take measures to restrain negative knowledge such as knowledge hidden activities, to activate the creativity of organization staff of intellectual resources. This paper contributes to innovation behavior literature which is an important part of innovation management based on both conservation of resources theory and social network theory.

## Introduction

Nowadays, the external environment is more and more dynamic, competitive and information-based, which puts forward higher and higher requirements for the rapid adaptability of enterprise organizations. Innovation has become an important means for enterprise organizations to obtain competitive advantages, and is the driving force for enterprise survival and development. As the main force of enterprise innovation, employees are the starting point and foundation of enterprise innovation, and are related to the survival and development of the organization. The knowledge, resources and abilities contained in employees are the main sources for enterprises to obtain sustainable competitive advantages. Employee innovation is the key factor for enterprises to improve their core competitiveness. How to effectively utilize their innovation potential, develop incentive mechanism and stimulate innovation behavior has gradually become the focus of enterprise managers and scholars.

Employee innovation behavior is a hot topic in the field of organizational management, and many scholars conclude it as employee out-of-role behavior, that is, the behavior that is not regulated by the organization but is formed spontaneously by employees and beneficial to the organization, which is the behavior performance beyond the requirements of job responsibilities (Niu and Liu, 2021). The innovation behavior of employees is the foundation of organizational productivity and efficiency and has always been a hot issue in academic circles. Scholars generally believe that effective interaction (Vila-Vázquez et al., 2020; Lai et al., 2021), positive atmosphere (Yihua et al., 2021), positive leadership (Hong and Jiahui, 2020; Ms et al., 2020; Yi and De-zhi, 2021) and other supportive and stable factors are important aspects of stimulating innovative behavior. With the rapid evolution of organizational diversification structure and the increasingly fierce competition in the workplace, conflicts of interest and interpersonal friction within the organization are inevitable, and workplace exclusion is becoming more and more common workplace problems (Ali et al., 2020; Howard et al., 2020). In general, most studies focus on the positive factors that influence employee innovation behavior. However, studies have found that negative factors have a stronger and more lasting effect on people than positive ones (Baumeister et al., 2001) Zhou, etc., (Zhao et al., 2016) scholars also suggest that the research on the impact of negative factors in organizations on employee innovation should be supplemented. workplace ostracism, as a form of workplace bullying, appears widely in the practice of organizational management. According to a survey by recruitment website, nearly 70 percent (67 percent) of white-collar workers surveyed said they had been ostracized at work. In the context of the increasingly service-oriented industrial structure, the rapid rise of the new generation of employees, and the increasingly diversified labor force, workplace ostracism can profoundly affect the organizational behavior of employees (Hitlan and Noel, 2009). In human resource management, organizational behavior and other research fields gradually attracted attention (Tao et al., 2021). It is obvious that workplace ostracism has a negative impact on employees’ innovation behavior. However, Chinese people always like “harmony” and attach importance to face, and there must be multiple influencing factors and complex influencing mechanisms between them. Therefore, this study intends to explore the mechanism of workplace ostracism and innovation behavior from a rational perspective.

From the perspective of *conservation of resources theory*, in order to maintain the balance of resources and prevent the loss of existing resources, individuals lacking resources will adopt defensive strategies to protect existing resources and prevent further loss of resources. Workplace ostracism will cause exhaustion and exhaustion of employees’ psychological, emotional and material resources. Workplace ostracism, as a passive interpersonal interaction, will affect the psychological state and behavior pattern of employees (Ferris et al., 2008). Employees will reduce their participation and concentration in work, and reduce their commitment, dedication, responsibility and emotional dependence on the organization and others (Chi and Liang, 2013). When ostracized employees perceive the loss of their existing resources, they will have a series of negative attitudes and retaliatory behavior patterns, thus inhibiting the generation of innovative ideas and the implementation of innovative behaviors. Based on the above theories and previous studies, this paper focuses on behavioral patterns and psychological states seeking mediators – knowledge hiding and organizational identification. Among them, knowledge hiding is an active negative interpersonal interaction to protect one’s own resources. Organizational identification refers to employees’ sense of identity and belonging to the organization. Meanwhile, social network theory (Granovetter, 1985) is also believed that employees, enterprises and their own resources are embedded in the existing network, and employees’ own behaviors are deeply affected by the complex interaction existing in the network. Based on this, the paper believes that when employees perceive rejection, they will engage in negative behaviors of knowledge hiding, which in turn will affect their network embeddedness in the organization, forming a vicious circle, thus reducing employees’ sense of organization identification and making them unwilling to contribute their innovative ideas or behaviors to the organization. Therefore, this paper constructs a chain mediation path to clarify the first key question through a complete mediation model, namely, what is the mechanism between workplace ostracism and employee innovation?

In the study of organizational operation process, interdependence stimulates frequent and extensive interpersonal communication, communication and cooperation among related employees in an organization (Ziyuan et al., 2014), is an important antecedent variable of innovation behavior. Studies have shown that high task interdependence means that employees’ work affects each other, which requires effective coordination among employees (Jinmei and Yong, 2014). By establishing common goals among employees, high task interdependence can enhance employees’ sense of responsibility, facilitate more effective communication, enable employees to obtain more decision-making information, and ultimately stimulate creative activities (Molm, 1994). Task interdependence creates a kind of interactive situation, which will have a profound impact on the self-regulation and behavior choice of individuals after they encounter workplace ostracism. Therefore, the second key question to be explored in this study is how task interdependence moderates the mechanism between workplace ostracism and knowledge hiding.

Current studies on workplace exclusion mainly focus on the impact of workplace ostracism on employee job burnout (Zhao and Liu, 2019), recommending behavior (Xia and Zhouli, 2014), knowledge sharing (Yuhong and Jianghua, 2020) and proactive behavior (Liang and Xiyuan, 2019; Qianlin et al., 2020),while few studies on the relationship between workplace exclusion and innovative behavior. Through the analysis of the literature related to employee innovation behavior, it can be found that there are few studies on the impact of workplace rejection and negative psychology on employee innovation behavior. This study attempts to analyze from the perspective of workplace exclusion and knowledge hiding, which have received less attention in the early stage. Review of the literature shows that organizational identification is beneficial to employee innovation behavior and has a positive impact on enterprise innovation activities. The study will focus on the association between workplace ostracism and innovation behavior and the role of knowledge hiding and organizational identification as mediators in their relationship, based on which the study also tests the moderating role of task interdependence.

On the other hand, in this study, conservation of resource theory and social network theory are used to construct a theoretical model to expand the application scope of the theory. Therefore, this investigation’s purpose is the empirical explanation of the association between workplace ostracism and innovation behavior and the role of knowledge hiding and organizational identification as mediators in their relationship in China. The study also finds task interdependence is a boundary condition of the direct association between workplace ostracism and knowledge hiding. This study will contribute to the existing literature by producing information about the causal relationship among workplace ostracism, knowledge hiding, organizational identification, innovation behavior and task interdependence. This study is also aimed to provide an empirical understanding of all these concepts in the context of Chinese company sector for the development of effective retention policies by the company.

The following paragraphs are a brief review of relevant literature and are followed up by the hypothesized relationships between focal constructs. Then the research methods, analyses, and findings are recorded and discussed. Finally, both the theoretical and managerial implications and the limitations and avenues for future research have been discussed.

## Literature review and hypothesis development

### Conservation of resources theory

Clinical psychologist Hobfoll put forward COR in 1989, emphasizing that individual resources are the core mechanism to explain the generation and response to stress. Initially, individual resources were broadly defined as all things that are valuable to individual survival and development, including objects, personal characteristics, conditions or energy resources that individuals value. COR’s core argument is that individuals will always strive to acquire, retain, protect, and cultivate the resources they value. Stress occurs when there is a potential or actual depletion of an individual’s resources. These attritions are profound, first because resources have instrumental value to people; Second, they have symbolic value, helping people define who they are. Thus, depletion of resources threatens positive self-perception. At this point, it is essential to use resistance to repair depletion, acquire resources and prevent further resource loss (Hobfoll et al., 2018).

### Workplace ostracism and employee innovation behavior

Workplace ostracism refers to the perceived neglect, exclusion or rejection of others in the workplace. From the perspective of organizational behavior and social psychology, this definition contains four meanings: first, workplace ostracism is a kind of “workplace cold violence” and “workplace cold treatment,” which mainly includes: mental abuse, psychological warfare, indifference between people, self-esteem injury, “wearing small shoes” and so on; Secondly, workplace ostracism is an individual’s subjective psychological feeling, and the severity of workplace ostracism perceived by employees varies with individual subjective evaluation. Thirdly, workplace ostracism represents the overall rejection feeling of employees. There is no clear and specific source of rejection. Generally speaking, the source of rejection may be their leaders, colleagues, subordinates or customers they come into contact with at work. Fourth, workplace ostracism refers to interpersonal ostracism in the workplace, which is a common workplace phenomenon. Workplace ostracism deprives employees of their right to be noticed and makes them feel less of their existence in the organization, bringing about “social death.” It not only makes individuals depressed and pessimistic, but also makes their basic needs unsatisfied, which ultimately affects employees’ behaviors in the workplace.

Innovation behavior refers to the behavior that individuals actively seek new ideas and innovations at work and carry out relevant practices and actions for the realization of new ideas, such as generating new innovations and providing new problem-solving methods, so as to help the organization improve performance (Janssen, 2000; Yun and Jintao, 2010). Individual innovation can drive organizational innovation. According to Hussain Awan et al. (2021), innovation climate is the driving force of organizational innovation and can promote the generation of employee innovation behavior. How to promote employee innovation behavior has become an important issue in management practice and academic discussion. Some scholars put forward that the generation of employees’ innovative behavior not only depends on external factors, but also is more influenced by employees’ internal psychological cognition (Zhiqiang et al., 2015). Some scholars believe that the generation of employees’ innovative behavior is closely related to their intrinsic motivation (Tu and Lu, 2013). Studies show that negative factors have as much influence on people as positive ones (Rujie et al., 2015). The interpersonal behavior in an organization is closely related to the individual work behavior of employees. Therefore, it is of great significance to explore the impact of workplace ostracism on employees’ innovation behavior for improving enterprises’ innovation capability.

As Faizan Gul et al. (2021) pointed out, when negative behaviors occur in the workplace, employees who are assaulted tend to engage in undesirable behavioral outcomes, such as knowledge hiding. According to the resource conservation theory, workplace ostracism is a subjective judgment based on employees’ self-perception, and the level of exclusion perceived by different employees constantly changes dynamically over the long term. In order to deal with similar stressful events, individuals will spend more energy and time to adjust their mentality and deal with workplace conflicts. However, putting all your energy into dealing with workplace stress can avoid long-term damage, but it can drain your resources if you have little effect in the short term (Xincai et al., 2021), as a result, individuals are too limited by time and energy to actively engage in extra-role activities of more beneficial organizations. In addition, because the creative activity itself has uncertainty and risk, staff from within the organization out, ignored or suppressed, they put forward creative ideas need to face more risk of rejection, leading to rejection employees more reluctant to have too many subjective initiative interactions, thus the workplace ostracism will weaken the intrinsic motivation of employees’ creativity (Xiuqing and Yanling, 2017). Based on the above analysis, this paper proposes the following hypotheses:

H_1_: workplace ostracism has a negative impact on innovation behavior.

### The mediating role of knowledge hiding

Making full use of employees’ knowledge assets can bring tremendous benefits to the organization, but the organization has no right to force employees to share knowledge with other members of the organization. Employees may be reluctant to share knowledge in order to maintain their status and value. Connelly et al. (2012) define this kind of behavior as knowledge hiding, that is, the behavior of concealing knowledge for a certain purpose in the face of others’ knowledge help. Knowledge hiding behavior is derived from the research of knowledge transfer and flow barriers in R&D team construction and management. Knowledge hiding can be manifested as evasive hiding, pretending to be deaf and dumb and rationalizing hiding. Knowledge storage behavior is common in organizations, which is mainly caused by employees’ hostility to knowledge sharing. The failure of knowledge sharing behavior in organizations is essentially due to employees’ hostility to knowledge sharing (Zhicheng et al., 2020), and knowledge hiding will further affect the level of knowledge contribution (Pengfei et al., 2022). Positive organizational knowledge culture has a negative impact on knowledge hiding behavior within the organization, and increasing team cooperation, trust and organizational commitment can reduce knowledge hiding behavior (Geofroy and Evans, 2017).

Research has shown that workplace ostracism (Tianru and Aizhong, 2019), experience of contradictory exchange relationship between superiors and subordinates (Shi et al., 2021), job insecurity (AIhua and Zisen, 2016) which are negative subjective feelings are positively correlated with employee knowledge hiding behavior. Workplace ostracism, as an important stressor of work situation, will increase the frequency and degree of knowledge hiding behavior Williams and Sommer (1997). According to the proposed “Need-threat Model,” when employees perceive the rejection of other members of the organization, individuals are isolated from the society or the organization they belong to, and forcibly cut off their contact with others. The four basic psychological needs of belonging, self-confidence, control and sense of existence will be significantly reduced. As Mohsin et al. (2022) pointed out, workplace ostracism has been shown to hide knowledge and feelings during the workday, as predicted by the COR theory. Attempts to hide valuable information are more likely to occur when employees are unaware that they may be punished for withholding sensitive data. According to the theory of resource conservation, in order to maintain the balance of resources and prevent the loss of existing resources, individuals lacking resources will adopt defensive strategies to protect existing resources and prevent further loss of resources. Psychological needs belong to personality trait resources. When employees lose their own resources, they will try their best to save other undamaged resources to make up for the damage caused by the loss of resources, so as to carry out knowledge hiding. In addition, conservation theory proposes that individuals experience tension when resources are depleted. In order to relieve tension and restore psychological balance, individuals respond by taking valuable resources from the environment to offset the depletion of resources. Based on the above analysis, this paper proposes the following hypotheses:

H_2_: workplace ostracism has a positive effect on knowledge hiding.

On the one side, based on the principle of reciprocity, when employees feel negative behaviors such as knowledge hiding, they will reciprocate with the same negative behaviors (such as knowledge hiding, etc.). Such a vicious cycle is not conducive to the innovation atmosphere of the organization. Knowledge hiding behavior will destroy the sense of trust among employees, reduce or reduce the efficiency of team cooperation, reduce communication among employees, and hinder the sharing of different views and knowledge among team members (Cerne et al., 2014), resulting in a vicious circle of mistrust, knowledge cannot be effectively spread and integration, is not conducive to employee innovation. On the other side, knowledge withholding entails an individual providing less information than the one required. Thus, it can be done intentionally since the person might not be sure that they are withholding important knowledge (Gul et al., 2021; Mohd et al., 2021).Employees who engage in knowledge hiding will not be willing to share their knowledge, which slows down the speed of knowledge accumulation and is not conducive to the generation of innovative behaviors. In order to maintain the balance of their own resources, the ostracized employees will have the psychology of knowledge hiding, and the knowledge hiding behavior among employees will inhibit the generation of their innovative behavior. Based on the above analysis, this paper proposes the following hypotheses:

H_3_: Knowledge hiding plays a mediating role between workplace ostracism and innovation behavior.

### The mediating role of organizational identification

Identification is an important way for individuals to obtain self-concept from the outside world. Identity is an important part of self-concept, which refers to “the extent to which an individual regards himself as an independent individual, a partner or a member of a group,” prompting an individual to perform the role behaviors expected by the organization (Fang, 2008). Patchen (1970) introduced it into organizational research and proposed the concept of organizational identity. Ashforth and Mael (1989) define organizational identity as a state in which individuals define themselves according to a specific organization membership or a perception of belonging to an organization. Organizational identity can help individuals answer the question of “who am I?” it emphasizes the process of integrating themselves with the organization in self-definition, from “I” to “we,” thus reflecting the dependence and belonging of individuals on an organization. When employees perceive negative behavior, their contribution to the organization is greatly reduced.

workplace ostracism, as a negative interpersonal interaction experience, leads to disharmony and distrust in employee relations, which may make employees feel that they are not accepted and recognized by the organization, thus reducing organizational identity. This paper holds that the impact of workplace ostracism on employees’ organizational identity is mainly reflected in two aspects. On the one hand, workplace ostracism undermines four basic needs of workers (Longzeng et al., 2010): Reduce the degree of organizational embeddedness of ostracized employees and harm their belongingness needs (Baumeister and Leary, 1995); It belongs to workplace cold violence, which makes excluded employees lose their sense of happiness and achievement at work, and destroys their self-esteem needs; Corrosion repels a sense of control over human-machine interaction and the surrounding environment, compromising its need for control (Friedland et al., 1992); It causes the “social death” of the excluded employees, depriving them of the meaning of their existence in the organization (Solomon et al., 1991). Employees who have been ostracized by the workplace for a long time will suffer from the loss of psychological and physical resources, which will affect their cognitive evaluation and emotion toward the organization and make them lack trust in the organization, thus affecting their sense of identity. On the other hand, workplace ostracism will weaken employees’ interpersonal communication, which is an important factor affecting organizational identity, so it will weaken employees’ organizational identity. Based on the above analysis, this paper proposes the following hypotheses:

H_4_: workplace ostracism has a negative impact on organizational identity.

As an individual psychological factor, intrinsic motivation is an important mediating variable of workplace ostracism affecting employee innovation behavior. Organizational identity is a subjective perception of employees, which can reflect the unity of employees and the organization. The results include cooperation, efforts and participation in decision-making beneficial to the organization (Ashforth et al., 2008). “Is actually similar to the employee’s” insider self-perception. Organizational identity is manifested by a high degree of employee-organizational goal matching, that is, employees are willing to complete the tasks delivered by the organization through strengthening self-learning and internal communication (Huafei and Zhang, 2020). Current studies have proved that organizational identity has a significant impact on employees’ innovation behavior (Ling et al., 2014). The higher the employee’s sense of identity to the organization, the more the employee will regard the interests of the organization as their own interests, link their own destiny with the organization, and actively improve work, and strive to improve work suggestions and innovative ideas; At the same time, organizational identity will strengthen the internal motivation of employees, and the insider identity becomes the motivation basis to motivate employees to do their best for the organization and the key to forming innovative behavior.

To sum up, this paper argues that employees who are ostracized in the workplace will reduce their organizational identity and thus inhibit their innovative behavior. Based on the above analysis, this paper proposes the following hypotheses:

H_5_: Organizational identity plays a mediating role between exclusion and innovation behavior in the workplace.

### The chain mediating role of knowledge hiding and organizational identification

Granovetter (1985) according to the proposed social network theory, individuals form various social networks by connecting with other organizations or institutions, and their own behaviors, especially economic behaviors, are deeply affected by the network, and the knowledge flow occurring in the network often exists in strong connections. According to the social network theory, when individual employees are embedded in the complex organizational network, they form a complex, interconnected and diversified network structure based on the knowledge flow as the carrier of knowledge. For individuals, important leaders and colleagues must be in the same organizational network structure. Therefore, in a specific organizational environment, individuals will feel pressure from network members when they take knowledge hiding behavior. From the perspective of self-verification, when employees feel pressure in the organization, they will think that they are not accepted by the organization, and it is difficult to form the perception of organizational identity. Driven by the need for self-consistency, employees will seek the surrounding evaluation to verify their negative self-concept, thus resulting in low organizational identity.

Combined with the hypothesis above, workplace ostracism has a positive effect on knowledge hiding, and knowledge hiding affects employees’ behavior through organizational identity. In other words, workplace ostracism will cause employees to feel the loss of their own resources, leading to their knowledge hiding. Employees who engage in knowledge hiding will feel the pressure from the organizational network in many aspects, reduce their organizational identity, make them lose their insider perception, and become reluctant to contribute innovative behaviors to the organization. Based on the above analysis, this paper proposes the following hypotheses:

H_6:_ Knowledge hiding and organizational identity. There is a chain mediating effect between workplace ostracism and innovation behavior, that is, workplace ostracism leads to knowledge hiding, reduces organizational identity, and hinders innovation behavior.

### The noderating role of task interdependence

Task interdependence refers to the structural connection between individual tasks and tasks of other members, such as target association, process cohesion, schedule coordination, resource sharing and allocation, and the degree of team cooperation requirements (Wageman, 1995). It reflects the extent to which team members depend on others for their work. The existence of task interdependence makes the interaction and cooperative behavior of team members become the basic characteristics of team operation. From the perspective of motivation information processing, task interdependence can provide opportunities for team members to share and process information, and enhance the accuracy of collective decision-making and the efficiency of team cooperation (Ziyuan et al., 2014). A job with a high degree of task interdependence is essential because of its relationships with other tasks. Employees have to communicate and collaborate with other relevant members to get the job done.

Task interdependence is not only a kind of work requirement, but also a kind of exchange criterion stipulated in the organization. It can provide a good exchange interaction environment for the organization, but also implies the requirement of mandatory exchange. According to social exchange theory, common value provides a common set of standards for complex exchanges in organizations, enabling all parties involved to exchange with the same situational definition. Therefore, in the context of high task interdependence, on the one hand, because the task itself is interactive, the excluded employees need to share the knowledge information or skills required by the work with other members in order to complete the work. On the other hand, task interdependence emphasizes the importance of employees’ joint efforts and cooperation, creates an atmosphere for information exchange and cooperation for the organization, and promotes information exchange among employees (Fong et al., 2018), weakened knowledge hiding.

To sum up, when task interdependence is high, employees have the responsibility to share job-related knowledge, and the organization provides an environment conducive to communication, employees cannot hide job-related knowledge, information or skills. When task interdependence is low, there is no communication and knowledge sharing can complete the task, even if the hidden knowledge, other colleagues also difficult to monitor or evaluate, have neither the knowledge sharing in the work force, the hidden knowledge is found that the risk of smaller again, this time whether to communicate to share depends entirely on personal will, in this case, In order to retain their own resource advantages, employees will choose knowledge hiding. Based on the above analysis, this paper proposes the following hypotheses:

H_7:_ Task interdependence moderated the positive effect of workplace ostracism on knowledge hiding.

**FIGURE 1:**
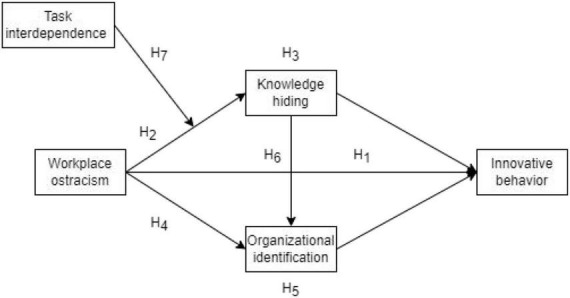
Theoretical research model.

To sum up, the research model is constructed, as shown in Figure 1.

## Materials and methods

### Research methodology

In order to ensure the balance and representativeness of sample data, the samples investigated in this paper are mainly enterprises from Inner Mongolia, Henan, Hubei, Fujian and Guangzhou. The survey samples of this study are mainly from enterprises in Internet, finance and other industries that have high requirements for employees’ innovation, and their employees are typical knowledge workers. Due to the epidemic, online questionnaire survey was adopted for data collection, and all questionnaire items were self-evaluated by employees. The questionnaire was distributed in two stages: pre-survey and formal survey.

The pre-survey is mainly for teachers with high professional level and management with many years of enterprise work experience. In the pre-survey stage, 122 valid data were collected by snowball method to analyze the reliability and validity of the initial questionnaire. The results showed that the Cronbach’s alpha coefficients of workplace exclusion, knowledge hiding, organizational identity, innovation behavior and task interdependence were all greater than 0.7, indicating good reliability of each scale. Through exploratory factor analysis and confirmatory factor analysis of the pre-survey data, some questions with unclear meanings, easy to cause ambiguity and poor reliability and validity are revised and deleted for many times according to the survey results, and the formal questionnaire is finally formed. In the formal investigation stage, questionnaire links are mainly sent through QQ, wechat and other platforms.

A total of 543 samples were collected in this survey, and 409 valid data were obtained after eliminating the samples with insufficient answer time, incomplete answers, consistent single or multiple variable options, and obvious rules or errors in answers, with an effective rate of 75.32%. The basic sample information is shown in Table 1.

**TABLE 1:** Basic information of samples.

Project	Category	The number	Accounted for (%)	Project	Category	The number	Proportion (%)
Gender	Male	207	51%	Education background	Junior College below	77	19%
	Female	202	49%		University degree	215	52%
Age	Under the age of 20	14	3%		A master’s degree	97	24%
	20 to 25 years old	68	17%		Dr.	20	5%
	25 to 30	105	26%	Working fixed number of year	the following 1 year	55	13%
	30–35 years old	98	24%		1–3 years	107	26%
	35–40 years old	88	21%		3 to 5 years	85	21%
	Above 40 years old	36	9%		5–10 years	74	18%
Job level	Ordinary employees	242	59%		10 years and above	88	22%
	Cadres at the grass-roots level	71	17%				
	Middle-level cadres	62	16%				
	Senior cadres	34	8%				

### Measures

In order to ensure the reliability and validity of the questionnaire, the following work was done in this study: First, all the scales used in this study were mature scales published in mainstream journals at home and abroad and verified in The Chinese context. All English scales are processed in a “translation-back” way to ensure semantic accuracy, and related items are screened, adjusted and repaired according to Chinese social and cultural background and characteristics of research objects. Second, in view of the sensitivity and concealment of negative behaviors such as workplace ostracism and knowledge hiding, as well as the deep-rooted guanxi and face values in local culture, all variables in this study are suitable for informal situations and are measured by anonymous self-assessment. At the same time, all indicators were measured by likert 5-point scale, with 1 being strongly disagree and 5 being strongly agree. The existing scales and measurement indicators are as follows.

(1) Workplace ostracism. The 10-item scale developed by Ferris et al. (2008) was adopted, such as “at work, some colleagues would ignore my views or feelings.” The Cronbach’s α coefficient of the scale was 0.917.

(2) Knowledge hiding. The measurement scale developed by Connelly et al. (2012) was adopted, with a total of 12 items. Typical items include “WHEN colleagues ask me for knowledge, I pretend not to know this information.” The Cronbach’s α coefficient of the scale was 0.929.

(3) Organizational identity. The organizational identity scale developed by Mael and Ashforth (1992) contains six questions, including “When others criticize my company, they criticize me.” The Cronbach’s α coefficient of the scale was 0.867.

(4) Task interdependence. Campion et al. (1996) compiled a measurement scale with 3 items in total. Typical items include “I cannot complete my task without other team members’ information and materials.” The Cronbach’s α coefficient of the scale was 0.897.

(5) Innovation behavior. Using Scott and Bruce (1994) developed a measurement scale with 6 items. Typical items include “the subordinate often puts forward creative ideas.” The Cronbach’s α coefficient of the scale was 0.874.

In addition, this paper selected four categorical variables as control variables based on the following reasons: First, according to previous studies, demographic variables such as gender, age and educational background can affect individual innovation behavior (Zhang et al., 2018; Yu and Bei, 2019); Second, studies have shown that female employees are more likely to be affected by events in the organization in terms of their emotions and behaviors (Warren and Catherine, 1999);Thirdly, previous studies on knowledge hiding show that both the characteristics of the employee subject and the organizational environment are important factors affecting the employee behavior (Baosheng and Qingpu, 2017).

## Results

### Reliability and validity test of questionnaire

Firstly, the reliability of each scale is tested by calculating the internal consistency coefficient of the scale. The results showed that the Cronbach’s α coefficients of workplace ostracism, knowledge hiding, organizational identity, innovative behavior and forgiveness were 0.917, 0.929, 0.867, 0.897, and 0.874, respectively, which were all higher than the minimum standard 0.7, indicating that the scale had good reliability.

Secondly, Amos 24.0 was used to test the convergence validity and structure validity among variables. According to the test results, the factor load of the 5 variables in the model corresponding to each topic is greater than 0.5, and the AVE values of workplace ostracism, knowledge hiding, organizational identity, task interdependence and innovation behavior are 0.526, 0.524, 0.520, 0.745, and 0.537, respectively. CR values were 0.917, 0.924, 0.867, 0.898, and 0.874, respectively, in which CR and AVE were all greater than 0.700 and 0.500, indicating that the scale had good convergence validity. The study further conducted a confirmatory factor test on the fitness of the variable model, and the results were shown in Table 2. Compared with other factor models, the goodness of fit of the five-factor model was the best, and x^2^/DF is less than 3, IFI, TLI, and TFI are all greater than 0.9, RMSEA and SRMR are less than 0.05, all of which are within the required range. Therefore, the research variables used in this study have good structural validity.

**TABLE 2:** Comparison of model fitness.

Model		Fitness index							
		
		χ^2^	df	χ^2^/df	IFI	TLI	CFI	RMSEA	SRMR
Five-factor model	(WO, KH, OI, TI, IB)	652.816	619	1.055	0.996	0.995	0.996	0.012	0.031
Four-factor model	(WO + KH, OI, TI, IB)	2398.017	623	3.849	0.77	0.753	0.769	0.084	0.112
Three-factor model	(WO + KH, OI, TI + IB)	3090.805	626	4.937	0.681	0.659	0.679	0.098	0.125
Two-factor model	(WO + KH, OI + TI + IB)	3666.311	628	5.838	0.607	0.581	0.605	0.109	0.134
Single factor model	(WO + KH + OI + TI + IB)	4571.849	629	7.268	0.489	0.457	0.487	0.124	0.142

WO stands for workplace ostracism, KH for knowledge hiding, OI for organizational identification, TI for task interdependence and IB for innovative behavior.

### Common method bias and confirmatory factor analysis

In this paper, two methods of program control and statistical control are used to avoid homologous variation bias. First of all, the same origin deviation should be avoided as far as possible in the questionnaire design and measurement procedure. In the scale design, this study protected the anonymity of questionnaire respondents by disrupting the order of questions and anonymous survey, and reduced their guessing of the measurement purpose. Secondly, the Harman single factor test was adopted, and the results showed that when all the items were subjected to unrotated principal component analysis at the same time, the explanation percentage of the first factor was lower than the threshold value of 40.0%, indicating that there was no serious common method deviation problem in the data. At the same time, according to the results of confirmatory factor analysis, the data fitting effect of single-factor model is significantly lower than the five-factor ideal, that is, the scale items involved do not belong to the same variable.

Since the single-factor test method may not be sensitive, the two-factor model is used to test, that is, the method factor is added as the global factor on the basis of the original factor (Honglei et al., 2014). If the method factor is added, the model fitting index optimization degree is very high, indicating the existence of serious common method bias. After testing, the improvement degree of CFI and TLI was less than 0.1, RMSEA and SRMR was less than 0.05, and the model was not improved effectively (χ^2^/df = 1.027, CFI = 0.998, TLI = 0.998, RMSEA = 0.008, SRMR = 0.030). The above test results indicate that the common method bias in this study is at an acceptable level and will not have a serious impact on the research results.

### Descriptive statistics of variables

Firstly, descriptive statistical analysis was conducted on each variable. Table 3 shows the mean value, standard deviation and correlation coefficient of each variable, among which gender, age, educational background and working years were the control variables. According to the results, workplace ostracism was significantly positively correlated with knowledge hiding (β = 0.304, *P* < 0.01), and negatively correlated with organizational identity (β = –0.241, *P* < 0.01) and innovation behavior (β = –0.338, *P* < 0.01). Knowledge hiding was negatively correlated with organizational identity (β = –0.249, *P* < 0.01) and innovation behavior (β = –0.374, *P* < 0.01), while organizational identity was positively correlated with innovation behavior (β = 0.466, *P* < 0.01).The results are consistent with the proposed hypothesis and provide a preliminary basis for further verification of the hypothesis.

**TABLE 3:** Descriptive statistical analysis of variables.

	1	2	3	4	5	6	7	8	9
1. Workplace ostracism	1								
2. Knowledge hiding	0.304**	1							
3. Organizational identity	0.241**	0.249**	1						
4. Innovative behavior	0.338**	0.374**	0.466**	1					
5. Task interdependence	0.272**	0.469**	0.176**	0.335**	1				
6. Gender	0.015	0.110*	0.018	0.05	0.012	1			
7. Age	0.034	0.069	0.021	0.074	0.042	0.113*	1		
8. Education	0.021	0.076	0.05	0.106*	0.019	0.017	0.184**	1	
9. Years of service	0.043	0.028	0.001	0.024	0.049	0.120*	0.641**	0.234**	1
The mean	2.877	3.048	3.382	3.298	2.873	1.490	3.700	2.150	3.080
The standard deviation	0.907	0.889	0.941	1.024	1.322	0.501	1.308	0.775	1.356

*** Means P < 0.001, ** means P < 0.01, * means P < 0.05.

### Test of direct effect and mediation effect

In this study, the hierarchical regression method in SPSS 26.0 was used to test the proposed hypothesis, and the relationship model between variables was constructed, respectively. Gender, age, educational background and working years were taken as control variables, and the results were shown in Table 4. According to Model 2, after controlling for variables, workplace ostracism is significantly negatively correlated with innovation behavior (β = –0.336, *P* < 0.001), assuming H_1_ Set up; According to model 6, workplace ostracism positively affects knowledge hiding (β = 0.300, *P* < 0.001), assuming H_2_ Verified; According to Model 3, knowledge hiding has a significant negative effect on innovation behavior (β = –0.287, *P* < 0.001). Meanwhile, workplace ostracism has a significant negative effect on innovation behavior, but the impact degree is lower than that of Model 2 (β = –0.250, *P* < 0.001). It shows that knowledge concealment plays a partial mediating role between exclusion and innovation behavior in the workplace was established. According to model 8, workplace ostracism has a significant negative impact on organizational identity (β = −0.240, *P* < 0.01), assuming H_4_Get support; According to Model 4, organizational identity has a positive impact on innovation behavior (β = 0.402, *P* < 0.001), while workplace ostracism has a significant negative impact on innovation behavior, but the impact degree is lower than that of Model 2 (β = −0.240, *P* < 0.001). It shows that organizational identity also plays a partial mediating role between workplace ostracism and innovation behavior was established.

**TABLE 4:** Results of hierarchical regression analysis of innovation behavior.

The variable name	Innovation behavior				Knowledge hiding		Organizational identification	
			
	M1	M2	M3	M4	M5	M6	M7	M8
Gender	0.046	0.039	0.009	0.034	0.111*	0.105*	0.018	0.013
Age	0.091	0.096	0.056	0.083	0.135*	0.140*	0.03	0.033
Education background	0.103	0.092	0.071	0.075	0.084	0.074	0.052	0.044
Working fixed number of year	0.065	0.050	0.011	0.041	0.148*	0.135*	0.033	0.022
Workplace ostracism		0.336***	0.250***	0.240***		0.300***		0.240***
Knowledge hidden			0.287***					
Organizational identification				0.402***				
R^2^	0.019	0.131	0.203	0.283	0.032	0.122	0.004	0.061
Δ R squared	0.009	0.120	0.192	0.272	0.023	0.111	0.006	0.049
F	1.904	12.166***	17.116***	26.410***	3.36*	11.211***	0.356	0.356***

*** Means P < 0.001, ** means P < 0.01, * means P < 0.05, and the table is the non-standardized coefficient.

The macro program of SPSS plug-in was used to further test the mediating effect between knowledge hiding and organizational identity on workplace ostracism and innovation behavior, and to verify the chain mediating effect of both. The Bootstrapping asymmetric confidence interval test method was used for testing, and repeated extraction was conducted for 5000 times with a confidence interval of 95%. The existence of mediating effect was verified by considering whether the confidence interval contained “0.” The results are shown in Table 5. The 95% confidence interval of the mediation path of knowledge hiding is [–0.120, –0.043], excluding 0, indicating that the mediation effect is significant further support; 95% confidence interval of organization identification with this mediation path is [–0.120, –0.035], excluding 0, indicating significant mediation effect, assuming H_5_ to be supported again; The 95% confidence interval of the chain mediation path “workplace ostracism → knowledge hiding → organizational identity → innovative behavior” is [–0.043, –0.010], excluding 0, indicating that knowledge hiding, organizational identity play a chain mediating role between workplace ostracism and innovative behavior, assuming H_6_ verified.

**TABLE 5:** Results of chain mediation effect test.

Mediating path	Effect of value	95% confidence interval	
		
		The lower limit	The higher limit
Workplace ostracism → knowledge hiding → innovative behavior	0.078	0.120	0.043
Workplace ostracism → organizational identification → innovative behavior	0.075	0.120	0.035
Workplace ostracism → knowledge hiding → organization identification → innovative behavior	0.024	0.043	0.010

### Test of moderating effect

**FIGURE 2:**
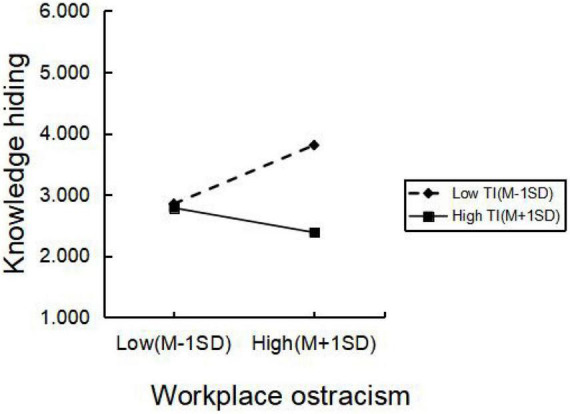
Modulation effect diagram.

Firstly, the correlation variables are centralized to reduce the interference of multicollinearity on the results. The process in SPSS was used to test the adjustment effect, and the results were shown in Table 6. The interaction item of workplace ostracism and task interdependence had a significant negative effect on knowledge hiding (β = –0.283, *P* < 0.001), indicating that task interdependence significantly moderated the relationship between workplace ostracism and knowledge hiding. In order to verify the regulation effect more intuitively, Aiken et al. (1991) were used in this paper. As shown in Figure 2: When task interdependence is low, workplace ostracism has a strong positive effect on knowledge hiding; When task interdependence was high, the positive effect of workplace ostracism on knowledge hiding was weakened. It indicates that when employees perceive high task interdependence, the positive effect of workplace ostracism on knowledge hiding is weakened, that is, the workplace ostracism of task interdependence has a negative moderating effect on knowledge hiding was established.

**TABLE 6:** Test results of moderating effect.

Variable	The coefficient of	t	p	95% confidence interval	
				
				The lower limit	The higher limit
Result variable: Knowledge hiding					
Workplace ostracism	0.153	3.869	0.000	0.075	0.231
Task interdependence	0.282	10.402	0.000	0.335	0.229
Workplace ostracism * Task interdependence	–0.283	9.547	0.000	0.342	0.225

R^2^ = 0.391, F = 86.729.

## Discussion

Our study aims to establish a relationship between workplace exclusion and innovative behavior. Existing researches mostly focus on the methods and approaches of stimulating positive activities such as innovation behavior, but ignore the influence mechanism of negative factors and countermeasures. Workplace exclusion, as a negative interpersonal interaction behavior, releases a signal of interpersonal disharmony (Mingyan et al., 2022). For this reason, our research decided to clarify the impact of negative factors such as workplace rejection on innovation behavior. Existing studies show that (Jianping and Tingzhong, 2020), due to the psychology of resource conservation, when employees feel more excluded in the workplace, they are less willing to engage in non-mandatory organizational behaviors. Our research also confirms that employees who experience workplace rejection are less likely to engage in innovative behavior.

The research also attempts to explore the mediating role between knowledge hiding, organizational identity and workplace exclusion and innovation behavior. Our research is also supported by the theory of resource conservation. The theory is that individuals strive to reduce the loss of their own resources. Exclusion in the workplace cuts off the connection between employees and the organization. In order to reduce the loss of their own resources, employees will hide their knowledge, which will aggravate the sense of resource deprivation of employees and thus reduce the occurrence of innovative behaviors. According to the resource conservation theory, resource depletion will cause “stress response” of employees (Jieqian et al., 2018). Workplace exclusion makes it difficult for employees to feel the connection with the organization, and reduces their sense of belonging and identity to the organization (Qingjin et al., 2020). As a result, the internal driving force of work is reduced, and the output of innovative behavior is weakened. The study also explains the chain mediating role of knowledge hiding and organizational identity between exclusion and innovation behavior in the workplace. Workplace rejection on the one hand, stimulate employee perceived organizational resources and negative psychological resources serious loss, on the other hand lead to employees never friendly hard working status for other high quality resources, resource depletion and resources is the relationship between the state further inspire the staff of the negative emotions, resulting in a retaliation hidden knowledge. However, knowledge concealment further intensifies the conflicts between organizations and reduces the identification degree of employees to the organization. Therefore, employees choose to reduce innovation behavior out of self-resource protection.

In addition, the moderating effect of task interdependence on workplace exclusion – knowledge hiding is also discussed. Specific tasks are the premise of team construction, and members will dynamically adjust the way of interaction with other members according to specific task situation (Tao et al., 2022). As for the variable task interdependence, existing studies (Jing and Xiaoli, 2021) mostly focused on its moderating effect, which is consistent with this study. When the task interdependence in the organization is higher, the organization has provided a situational atmosphere for employees to exchange. In this situation, knowledge hiding is no longer secret and convenient, and is likely to be found and regarded as an irresponsible behavior. In order to complete the work normally, Even if employees perceive workplace rejection, they have to reduce their knowledge hiding behavior to reduce the risk of their “illegal” behavior being discovered.

## Theoretical contribution

This paper conducts a systematic empirical study on the influence and motivation mechanism of workplace ostracism on various dimensions of innovation behavior in the Context of China, which has important theoretical significance, mainly reflected in:

(1) This study enriches the existing research results on workplace ostracism and employee innovation behavior. In the past, few literatures systematically discussed how workplace ostracism affects employee innovation behavior. Existing researches mostly focus on positive organizational climate, situation or psychological state, such as organizational innovation climate, leadership style and positive emotions, which are conducive to employee innovation behavior. However, as a negative situational factor in the organization, workplace ostracism has little influence on employee innovation behavior. Through the research on workplace ostracism, it is found that the innovation behavior of employees is an active behavior, and the inhibitory effect of workplace ostracism on it is consistent with the negative effect of workplace ostracism on active behavior. This study from the antecedent of explore employee innovative behavior, analyzes the workplace ostracism caused by violation of reciprocity staff concealed create internal mechanism of the behavior or thoughts, help to open the workplace and employee innovative behavior of the relationship between “black box,” added to the composite model of rejection in the workplace and employee innovative behavior of this variable.
(2) This study uses resource conservation theory and social network theory to analyze the internal mechanism among key research variables and construct the overall logic among all research variables, so as to extract different connotations of the same variable and reveal the influence mechanism of workplace ostracism on innovation behavior more comprehensively and completely. At the same time, the application of social network theory in the field of organizational behavior is broadened. Most of the previous studies only focused on the single connotation of variables. On the one hand, the present study verified the independent mediating effect between knowledge hiding and organizational identity on workplace ostracism and innovation behavior through resource conservation theory, and reflected the loss of relationship resources through the occurrence of knowledge hiding and the reduction of relationship identity. On the other hand, guided by the social network theory, from the perspective of self validation, the chain between the workplace and innovation behavior is explained the validity of mediation, when employees behavior affected by the degree of the embedded network, when the knowledge hidden feeling the pressure from other subject network, thus reducing its organizational identification, According to different theories, different connotations of the same variable are extracted to capture the internal relationship between individual network state change and resource gain and loss, and then the influencing mechanism between workplace ostracism and innovation behavior is more completely explained from the internal mechanism of variables and the overall logic of the model. At the same time, this study also makes social network theory become a new theoretical basis for exploring the consequences of workplace ostracism or the antecedents of innovative behavior, and broadens the application of this theory in the field of organizational behavior.
(3) By examining task interdependence as a boundary condition, this study explains the moderating effect of workplace ostracism – knowledge hiding, and extends social exchange theory from the perspective of situational factors. Compared with previous attempts to explain the phenomenon that employees have different behaviors in the same situation by exploring individual differences of employees or differences in their perceived atmosphere, this study specifically analyzes whether work characteristics will also cause behavioral differences of employees through task interdependence, a situational factor. This study suggests that task interdependence is a particularly important special situational variable, which can effectively stimulate individuals to improve their self-control and regulation ability, and buffer the negative effects of workplace ostracism on employees’ cognition and behavior. Therefore, the moderated model constructed in this study essentially verifies the impact of the interaction between situational factors and workplace negative behaviors on the individual behaviors of employees, which is helpful to further understand the differences and relationships among different constructs contained in the theory.

## Practical implications

Stimulating employee innovation can make enterprises develop in a competitive market environment. Workplace ostracism, as an important situational factor affecting employee behavior, will reduce employee innovation behavior and is not conducive to the long-term development of enterprises. Organizations and managers should prevent and reduce the harm caused by workplace ostracism in management practice. The results of this study have the following management implications:

(1) Enterprises should pay attention to the negative impact of workplace ostracism, focus on the leading factors of workplace ostracism, build an inclusive corporate organizational culture, and create a good team atmosphere. At present, organizational culture has been proved to be one of the important means to promote organizational knowledge sharing (Ming and Jianming, 2009). On the one hand, managers should adhere to the “people-oriented” management concept and build an encouraging, inclusive, open and innovative corporate organizational culture. Managers should advocate members’ pursuit of collective interests to reduce the individualistic cultural atmosphere in the organization; Identify the immature ideas generated in the process of innovation so as to enhance members’ sense of value and enhance their sense of organizational identity; By delegating power to the leader, strengthening authorization and other means to shorten the management level in the enterprise to improve the atmosphere of knowledge sharing in the enterprise. On the other hand, the negative effects of workplace ostracism can be weakened by designing reasonable work processes to improve the degree of task interdependence of employees and constructing a reciprocal social exchange atmosphere. Through job design, employees can actively improve the degree of task interdependence, increase the opportunities for communication and communication among employees, and promote mutual understanding among colleagues. Meanwhile, highly related work tasks can objectively reduce employees’ knowledge hiding behavior and reduce the harm caused by workplace ostracism.
(2) Enterprises should combine rigidity and softness, combine strict management with humanistic care, and construct scientific human resource system. On the one hand, during recruitment or training, enterprises can conduct psychological tests on employees to understand their personality tendencies and carry out targeted training for employees according to their personality characteristics, so as to correctly understand and master the skills to cope with workplace ostracism. For example, employees with high social self-efficacy are less likely to suffer from workplace ostracism. Therefore, enterprises need to pay more attention to psychological counseling and organizational care for employees with low social self-efficacy. On the other hand, enterprises should establish communication and feedback mechanisms and platforms for employees. Establish diversified feedback channels so that excluded employees can find feedback channels in time to seek help and coordination from the organization and reduce the cost of feedback for employees; Formulate corresponding enterprise systems or build knowledge sharing platforms to encourage employees to share knowledge, such as relevant reward systems, the establishment of diversified dialogue platforms and communication channels within the enterprise, so as to reduce knowledge hiding behaviors caused by colleague rejection.
(3) Enterprise managers should adopt various ways to create communication opportunities for employees, encourage self-resolution or assist employees to adjust negative emotions through a third party, and improve employees’ identification with the organization. First, the organization should improve similar symposium of formal communication channels, or by setting the break room, lounge areas and other places of ways for employees to create informal communication, complement each other, facilitate prompt employees through communication workplace reject rational recognition, self resolve daily friction or misunderstanding, offset by depletion of the resources. Second, the organization shall be given according to the actual situation for HRBP team to new role, not only through HRBP strategy to make the human resources department personnel to participate in business management and operation, at the same time encourage in-depth business department of human resources management psychological mediator role, to a third party position deep communication with business department staff, to help employees get rid of emotional distress, Avoid the vicious cycle of workplace ostracism. This can not only reduce the psychological pressure for employees to seek help when they are rejected by colleagues, but also gradually curb the generation of rejection in the long-term cultural influence.

## Research limitations and future prospects

This study is a preliminary exploration of the workplace ostracism and innovation behavior of employees in enterprises in the Context of China, which has certain reference value for human resource management and employee innovation motivation in enterprises, but the research has certain limitations.

First of all, this research adopts the workplace ostracism, hidden knowledge, organizational identification and task interdependence scale is based on the development of the background of western organization, although in the study, through the strict translation – translation to program, and through the preliminary investigation and so on a variety of ways to minimize the item ambiguity and program error, but will be developed on the basis of foreign situation.

There may be some limitations in the application of the scale. In the future, relevant scales can be revised or developed based on the unique organizational environment in China to reflect the similarities and differences of the same variable under the influence of different cultures.

Secondly, the data used in this study are the sample data of the questionnaire, but the questionnaire data are all filled in by the same subject at the same time, which is subjective to a certain extent. Therefore, the research content of this paper can be expanded and improved by combining interviews, pairing and other methods in future research. At the same time, the cross-sectional data used in this paper cannot obtain the dynamic influence process between variables. Future research should consider using longitudinal tracking method with time span to explore the mechanism of the interaction between workplace ostracism and innovation behavior.

Finally, this study mainly explores the influence mechanism between workplace ostracism and innovation behavior from the individual level, and verifies the mediating effect of two important factors, behavioral pattern and psychological state. But in fact, it should be a multi-layer, multi-factor complex process. In the future, other mediating variables that have not been paid attention to in this study can be explored from the team level and the organization level, and the theoretical research results can be improved from multiple perspectives, such as the influence of factors such as team differential atmosphere and belonging need on workplace ostracism and innovation behavior.

## Conclusion

Our study aims to establish the relationship between workplace ostracism on innovation behavior. Using the conservation of resource and social network theory, discusses the workplace ostracism on the inner mechanism of innovation behavior and boundary conditions, and the inspection of hidden knowledge, organizational identity intermediary role and task interdependence adjust action, through a structural equation model to collect 409 samples from all over the country to the empirical analysis, Some conclusions with theoretical and practical value are obtained. First, we verify previous studies that workplace exclusion has a significant negative effect on innovation behavior.

## Data availability statement

The original contributions presented in this study are included in the article/supplementary material, further inquiries can be directed to the corresponding author.

## Ethics statement

Written informed consent was obtained from the individual(s) for the publication of any potentially identifiable images or data included in this article.

## Author contributions

YL contributed to conception and design of the study. YX performed the statistical analysis and wrote sections of the manuscript. YL and YX wrote the first draft of the manuscript. Both authors contributed to manuscript revision, read, and approved the submitted version.
